# Bioactive Plasmid- and Phage-Encoded Antimicrobial Peptides (AMPs) in the Human Gut: A Metatranscriptome–Virome Profiling Reveals Exploratory Links to Metabolic Human Diseases

**DOI:** 10.1007/s00248-025-02620-2

**Published:** 2025-11-28

**Authors:** Luigui Gallardo-Becerra, Fernanda Cornejo-Granados, Shirley Bikel, Iván Arenas, Gamaliel López-Leal, Carolina Alvarado-Gonzalez, Filiberto Sánchez-López, Rubiceli Manzo, Gerardo Corzo, Gerardo P. Espino-Solis, Samuel Canizales-Quinteros, Adrian Ochoa-Leyva

**Affiliations:** 1 Departamento de Microbiología Molecular, Instituto de Biotecnología, Universidad Nacional Autónoma de México, Avenida Universidad 2001, C.P. 62210 Cuernavaca, Morelos Mexico; 2 Departamento de Medicina Molecular y Bioprocesos, Instituto de Biotecnología, Universidad Nacional Autónoma de México, 62210 Cuernavaca, Morelos Mexico; 3 Laboratorio de Biología Computacional y Virómica Integrativa, Centro de Investigación en Dinámica Celular, Universidad Autónoma del Estado de Morelos, 62209 Cuernavaca, Mexico; 4 Facultad de Medicina y Ciencias Biomédicas, Laboratorio Nacional de Citometría de Flujo, Autonomous University of Chihuahua, Circuito Universitario S/N, Campus II, 31125 Chihuahua, Mexico; 5 Facultad de Química, UNAM/Instituto Nacional de Medicina Genómica (INMEGEN), Unidad de Genómica de Poblaciones Aplicada a La Salud, Mexico City, Mexico

**Keywords:** Antimicrobial peptides, AMPs, Phages, Metatranscriptome, Virome, Plasmid

## Abstract

Microbe-derived antimicrobial peptides (AMPs) can shape gut community structure; however, their contribution to disease-associated dysbiosis remains poorly understood. We assembled fecal metatranscriptomes from individuals with normal weight (NW), obesity (O), and obesity with metabolic syndrome (OMS), yielding 51,087 non-human transcripts. We screened 1,095 small open reading frames (smORFs) using AMP-prediction algorithms combined with stringent post-hoc bioinformatics filters identifying 51 high-confidence AMP candidates. Most matched bacterial homologs, predominantly *Faecalibacterium prausnitzii*, while eight mapped to plasmids or bacteriophages. Differential expression identified two and four AMPs overexpressed in O and OMS, respectively. Two of them were originated from chromosomes, three from phages, and one from plasmid. Notably, the over-expression of these AMPs was negatively correlated with healthy-associated bacteria and positively correlated with obesity-enriched taxa. Furthermore, these AMPs were broadly detectable across 372 external gut metatranscriptomes (prevalence up to 94% of the samples) indicating conservation within the human gut microbiome and highlighting mobile elements as an overlooked reservoir of transcriptionally active AMPs. Using DNA virome sequencing and prophage analyses, we suggested phage origin of the transcribed AMPs. We further synthesized a phage-encoded AMP (AMP-3020), demonstrating broad-spectrum activity against Gram-positive and Gram-negative bacteria, without detectable cytotoxicity toward human immune T cells. This supports the idea that phages could encode functional AMPs capable of shaping gut community structure by suppressing diverse bacteria without harming host immune cells. Our gut metatranscriptome–virome profiling revealed a conservative core of actively transcribed, plasmid- and phage-encoded AMPs with exploratory associations to obesity/MetS. These findings support mobile-element AMPs as candidate ecological regulators and motivate validation in larger cohorts and mechanistic models.

**Supplementary Information:**

The online version contains supplementary material available at 10.1007/s00248-025-02620-2.

## Introduction

Childhood obesity has emerged as a critical public health challenge [1], significantly increasing the risk of type 2 diabetes, cardiovascular diseases, and non-alcoholic fatty liver disease in adulthood [2]. It also predisposes to metabolic syndrome (MetS) [3], characterized by high blood glucose levels, elevated triglycerides, low high-density lipoproteins (HDL) levels, and hypertension [4, 5]. Together, these abnormalities diminish quality of life and increase the risk of disability and premature mortality [2, 6]. The situation is particularly alarming in Mexico, where nearly one in five school-aged children is obese and many likely have undiagnosed metabolic syndrome (MetS) [7], underscoring the urgent need for effective preventive and therapeutic strategies.

A growing body of work links obesity to shifts in gut microbial communities, as demonstrated by 16S rRNA profiling, shotgun metagenomics, and metatranscriptomics [8]. Viromics further implicates bacteriophages as key modulators of these communities, which can either maintain a healthy microbe balance (eubiosis) or induce an imbalance (dysbiosis) [9]. Dissecting microbe-phage interactions may enable microbiome-targeted strategies to prevent and treat obesity and metabolic syndrome.

Bidirectional communication between hosts and their resident microbes is mainly mediated by the "secrebiome," a collection of proteins released into the environment [8]. This includes enzymes, toxins, and antimicrobial peptides (AMPs), which are produced by both the host and microbiota as a key innate defense strategy [10, 11]. Despite their small size, AMPs exhibit diverse antimicrobial activities, from membrane disruption to binding essential intracellular targets [12]. In bacteria, AMPs can be synthesized through the proteolytic processing of larger precursor proteins [13, 14], non-ribosomal peptide synthetase (NRPS) pathways [15], and dedicated genomic genes [16]. Recent metagenomic studies have highlighted small open reading frames (smORFs; < 100 codons), as a rich reservoir of novel microbiome-derived AMPs [17–19]. Early large-scale surveys of human microbiomes uncovered thousands of previously unannotated smORFs, some with putative antimicrobial functions [19]. Understanding the diversity and regulation of these smORF-encoded AMPs is crucial for elucidating host-microbiome interactions in health and disease. In the human gut, host-encoded AMPs act as gatekeepers, regulating opportunistic pathogens and the composition of the resident microbiota [20, 21]. They help maintain microbial diversity, which is essential for intestinal health, and they are naturally compatible with the host due to their co-evolution within the intestinal ecosystem [22–24]. However, some AMPs may increase susceptibility to viral infections, underscoring context-dependent effects [25]. At the same time, microbial-derived AMPs foster competition among bacteria, allowing AMP-producing species to secure ecological niches and influence microbial dynamics [26]. Horizontal gene transfer via plasmids and bacteriophages (phages) expands AMP repertoires and disseminates AMP-encoding genes, enhancing bacterial fitness [27–30]. However, no study has established a link between AMPs and the dysbiosis seen in disorders like obesity and metabolic syndrome.

Metatranscriptomics provides insights into which AMP genes are actively transcribed in situ, highlighting those involved in community self-regulation. This study examines gut metatranscriptomes from healthy, obese, and obese with metabolic syndrome individuals to catalog differentially expressed AMPs and explore their roles in microbiota dysbiosis. To our knowledge, this study is among the first to connect the actively transcribed gut AMP repertoire with obesity and metabolic syndrome, laying groundwork for microbiome-targeted interventions.

## Materials and Methods

### De Novo Assembly and AMP Prediction

We analyzed RNA-seq data from two normal-weight (NW), three obese (O), and three obese children with Metabolic Syndrome (OMS), as detailed in a prior study (BioProject PRJNA600247) [8]. Quality assessment was performed using FastQC (https://www.bioinformatics.babraham.ac.uk/projects/fastqc/), followed by filtering with a Q20 Phred score and trimming using Trimmomatic v0.36. We removed sequencing adapters, ambiguous bases and rRNAs using Ribopicker v0.4.3 and the SILVA rRNA database (138 release). The remaining non-rRNA sequences were aligned to the human genome and transcriptome with Kneaddata to eliminate human derived sequences (https://github.com/biobakery/kneaddata). The remaining sequences were then used as input for the de novo transcriptome assembly with the Trinity assembler, adjusting to conserve small transcripts. After that, the original reads were aligned to the transcriptome using Bowtie2. Expression levels were calculated by Expectation (RSEM). After, we removed the transcripts with an expression below 1 in any sample or with less than three cumulative observations across all samples. Then, we employed TransDecoder (https://github.com/TransDecoder/) to identify ORF candidates within protein-coding regions of the transcript sequences, adjusting its parameters to obtain the smORFs (-m 5). The small proteins derived from these smORFs were analyzed for AMP prediction using Macrel (https://github.com/BigDataBiology/macrel), AxPEP (https://sourceforge.net/projects/axpep/), and AMP Scanner V2 (https://www.dveltri.com/ascan/v2/ascan.html) with default parameters.

### Taxonomic Classification, Phylogeny, Genomic Synteny and Prophage Analyses

The transcript sequences encoding AMPs were used for homology search against NCBI’s NT database using the BLASTN algorithm with the following parameters: e-value ≤  0.00001, identity  ≥80%, and coverage  ≥80%. The associated taxonomy was determined with a Last Common Ancestor (LCA) using MEGAN6 Community Edition (v.6.25.10). The genomic context was obtained for all transcripts, using BLASTN best-hit, and the phylogenic trees were created with iTOL (https://itol.embl.de). Plots for genomic context were created with the AnnotationSketch drawing library (https://genometools.org/annotationsketch.html). The genomic synteny comparisons were conducted using Easyfig (https://mjsull.github.io/Easyfig/) with the BLASTN algorithm. We highlighted genomes containing the AMPs identified in this study in bold in the corresponding figure. Arrows indicate the positions of coding sequences, while shaded lines represent the degree of homology between genomic regions or genomes. We assigned functional categories based on the COG classification system using the COG classifier tool (https://github.com/moshi4/COGclassifier), with each category represented by a distinct color to facilitate interpretation. We screened the bacterial genomes with VirSorter2 to determine whether the AMPs we identified were embedded within prophage regions (https://github.com/jiarong/VirSorter2).

### Read Mapping of Viral DNA to Phage Genomes

The quality-filtered phageome reads were obtained from a previously published dataset by our laboratory, which was derived from the same cohort (BioProject: PRJNA646512). We merged all R1 files into a single R1 file and all R2 files into a single R2 file to create a representative population of the entire virome. Next, we created an index for each phage genome using bowtie2-build with default parameters. We aligned the merged reads to the bacteriophage genomes using Bowtie2 with the parameters ‘—no-unal –end-to-end –very-sensitive.’ Finally, we calculated the total genome coverage and the X-fold coverage for each genome.

### 16S Microbiota Profiling

We obtained the V4 region of 16S rRNA gene for the samples from a previously published dataset by our laboratory (BioProject PRJNA600247) [8]. All quality-filtered sequences were joined and analyzed using QIIME2 (v2024.5). Briefly, raw sequences were imported and dereplicated, followed by de novo clustering at a 97% identity threshold. Taxonomic classification was performed using a Naive Bayes classifier trained on the SILVA 138 reference database. Taxonomy bar plots were generated, and amplicon sequence variants (ASVs) that were present in more than half of the samples (n = 4) were retained for Spearman correlation analysis against AMP abundances.

### Peptide Synthesis and Purification

ADR1 and ADR2 were chemically synthesized by a solid-phase method using the Fmoc methodology (GenScript Biotech, Piscataway, NJ) and purified. Briefly, ten milligrams of crude synthetic peptides were dissolved in one milliliter of 20% aqueous acetonitrile solution and were separated each by reverse phase HPLC on an analytic C18 column (Zorbax SB-C18, Agilent, USA). The C18 column was equilibrated in 20% aqueous acetonitrile containing 0.1% TFA, and the synthetic peptides were separated using a linear gradient of acetonitrile/0.1% TFA from 20 to 60% in 40 min at a flow rate of 1 mL/min. The presence of each peptide was monitored at 220 nm. The molecular mass of each peptide was determined by mass spectrometry.

### Antibacterial Assays

The assessment of bacterial growth was conducted using a broth microdilution assay according to the Clinical and Laboratory Standards Institute guidelines. The reference strains, *Pseudomonas aeruginosa* (ATCC 27853), *Klebsiella pneumoniae* (ATCC700603), *Staphylococcus aureus* (ATCC 29213) and *Streptococcus pneumoniae* (ATCC46916) were cultured in Mueller–Hinton broth (MHB) at 37ºC during overnight incubation. Following this culture period, the samples were diluted in MHB to achieve an endpoint corresponding to an absorbance between 0.08 and 0.13 units at 625 nm, followed by a further dilution of 1:100 in MHB (approximately 1 × 108 CFU/mL). An aliquot of fifty microliters from each bacterial suspension was introduced into each well of a 96-well microtiter culture plate, which contained 50 µL of MHB supplemented with varying concentrations of the synthetic peptides ADR1 and ADR2 (100, 50, 25, 12.5, 6.2, and 3.13 µg/mL). The growth of each bacterial strain was quantitatively evaluated by measuring absorbance at a wavelength of 630 nm after an incubation period of 18 h at 37 °C.

### Flow Cytometry Analysis of T Cell Populations

Human red blood cells were collected from a healthy donor who gave verbal consent for phlebotomy. This related experiment was approved by the Ethics Committee of the Facultad de Medicina y Ciencias Biomédicas of the Universidad Autónoma de Chihuahua with registration number CI-068–19**. **To assess the potential cytotoxic effects of the synthetic peptides on human immune cells, peripheral blood mononuclear cells (PBMCs) were isolated from three healthy donors. The cells were cultured in RPMI 1640 medium (GIBCO, 11875–085, Paisley, SCT, UK) supplemented with 5% fetal bovine serum (FBS) (GIBCO, 26140–079, Grand Island, NE, USA) and GlutaMAX (GIBCO, 35050–061, Grand Island, NE, USA). PBMCs were seeded in 96-well plates and exposed to each AMP peptide (ADR1 and ADR2) at 20 mg/mL. Cells cultured in medium alone were used as untreated controls. The initial evaluation was performed one hour after peptide exposure to determine the immediate effects on T lymphocyte populations. Subsequently, the PBMCs were incubated overnight at 37 °C in a humidified atmosphere with 5% CO₂ to assess potential delayed or sustained effects. Following incubation, cells were stained with the BD TriTest™ reagent (Becton Dickinson and Company, BD Biosciences, San Jose, CA, USA) to identify CD3⁺ T cells and their CD4⁺ and CD8⁺ subpopulations, the viability of CD3⁺, CD4⁺, and CD8⁺ T cell subpopulations was evaluated using the LIVE/DEAD™ Fixable Near-IR Dead Cell Stain Kit (Invitrogen™, Thermo Fisher Scientific, Eugene, OR, USA). The absolute number of cells was acquired using BDTM Liquid Counting Beads (BD Biosciences). Flow cytometric analysis was performed using a FACS Canto flow cytometer (BD Biosciences), and the data were analyzed with FlowJo software (BD Biosciences). Graphical representations and statistical analyses were performed using GraphPad Prism (GraphPad Software, San Diego, CA, USA). This experimental design allowed for the evaluation of peptide-induced changes in the distribution and viability of key T lymphocyte subsets, providing insight into the potential immunotoxicity or immunomodulatory properties of the peptides in human immune cells.

## Results

### Identification and Taxonomic Classification of AMP-Encoding smORFs of the Gut Microbial Transcripts

**Fig. 1:**
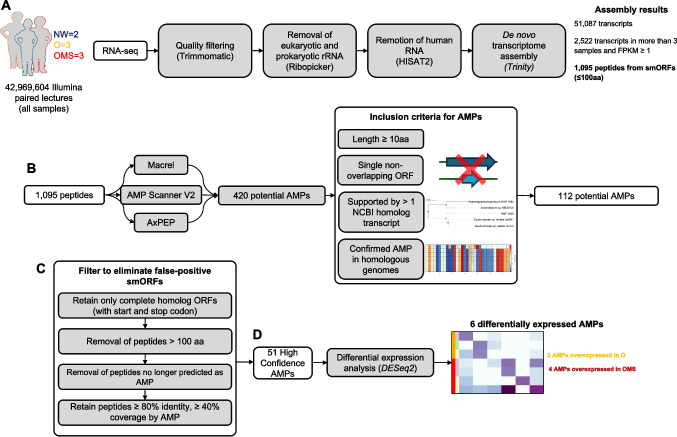
Workflow for identifying gut-expressed AMPs from fecal metatranscriptomes. **A** Data Processing. Raw Illumina reads were quality filtered and depleted of host and rRNA sequences. The remaining reads were de novo assembled, and smORFs were extracted. **B** AMP discovery. smORFs were screened using three independent predictors Macrel, AMP-Scanner v2, and AxPEP. Candidate AMPs undergo manual curation considering peptide length, genomic context, transcript support, and protein homolog evidence. **C** False-positive filtering of smORFs. Curated AMP candidates were further filtered to remove likely spurious smORFs. **D** Differential Expression. High-confidence AMPs were transcriptionally quantified and compared between obesity (O) and obesity with metabolic syndrome (OMS), yielding the final set of disease-associated, gut expressed AMPs

We analyzed fecal metatranscriptomes from a previously characterized pediatric cohort (BioProject PRJNA600247) [8], comprising two normal-weight (NW), three obese (O), and three obese children with metabolic syndrome (OMS) (Fig. 1A). After read-quality control and removal of eukaryotic and prokaryotic ribosomal RNA and human RNA sequences, 42.97 million high-quality paired-end reads remained. De novo assembly with Trinity yielded 51,087 transcripts (N50 = 1,372 bp) and 36.22 million assembled nucleotides (Supplementary Tables 1–2 and Supplementary Fig. 1).Of these, 35,803 transcripts encoded smORFs of less or equal than 100 amino acids. Transcript abundance was recalculated as Fragments per Kilobase of transcript per Million mapped reads (FPKM), and we retained transcripts with an FPKM ≥ 1 present in ≥ 3 samples, resulting in 1,095 reproducibly expressed transcripts (Fig. 1A).

We assessed the antimicrobial potential of 1,095 smORF-encoded peptides using three prediction tools Macrel [31], AxPEP [32], and AMP Scanner V2 [33]. This screening identified 420 potential AMPs supported by a least one tool. We retained only AMPs that met all three criteria: (i) length > 10 amino acids; (ii) encoded by a single, non-overlapping ORF (no overlap with any other coding sequence); and (iii) supported by at least one homolog transcript in NCBI (Fig. 1B). After filtering, 112 AMP candidates remained (Fig. 1B). To mitigate potential overestimation of small ORFs, given their limited prevalence in bacterial genomes [19], we scrutinized our initial set of 112 AMPs and found that several lacked start and/or stop codons in their original transcripts, consistent with possible false positives AMPs. We therefore applied a stringent post hoc filter (Fig. 1C): (i) selected peptides from homologous transcripts with clearly defined start and stop codons; (ii) excluded peptides > 100 aa; (iii) removed sequences no longer predicted as AMPs; and (iv) retained only homolog peptides for which our AMP aligned with > 80% sequence identity and > 40% coverage. After applying these criteria, the AMP set was reduced from 112 to 51. Notably, the retained AMPs align to their reference peptides with a mean coverage of 92% and a mean sequence identity of 97%, indicating that only small N- or C-terminal fragments were typically missing (Supplementary Table 3). All homologous genes in NCBI were annotated as hypothetical or uncharacterized. Consistent with this, our BLAST analyses of the 51 AMP peptide sequences returned only homologs labeled with unknown function. This suggest that these proteins lack prior functional assignments and that our results provide a putative antimicrobial role for this set. From the final 51 AMPs, AxPEP predicted 36 peptides (70.6%), AMP Scanner v2 predicted 23 (45.1%), and Macrel prediected 5 (9.8%). Only three peptides were unanimously predicted by three tools (Supplementary Fig. 2A).

To contextualize predictor performance, we compiled a reference panel of 1,390 experimentally validated microbe-derived peptides from APD3 [34], dbAMP [35], and DRAMP [36], confirming 1,332 (95.8%) as bona-fide AMPs. Benchmarking against this panel highlights substantial inter-tool variability and supports the importance of utilizing multiple predictors for candidate selection.

Taxonomic assignment of the 51 AMP-encoding transcripts indicated that 44 (86.3%) were bacterial, with *Faecalibacterium prausnitzii* accounting for 16 transcripts (31.4%) (Supplementary Fig. 2B). The remaining 7 transcripts (13.7%) were linked to the gut virome, mainly comprising Caudovirales, including Myoviridae and Siphoviridae members. This underscores bacteriophages as an additional source of AMP genes. Notably, one transcript was associated with a plasmid, highlighting the contribution of mobile genetic elements to the gut ecosystem AMP repertoire.

### Widespread Transcription of AMPs Differentially Expressed in O and OMS

**Fig. 2:**
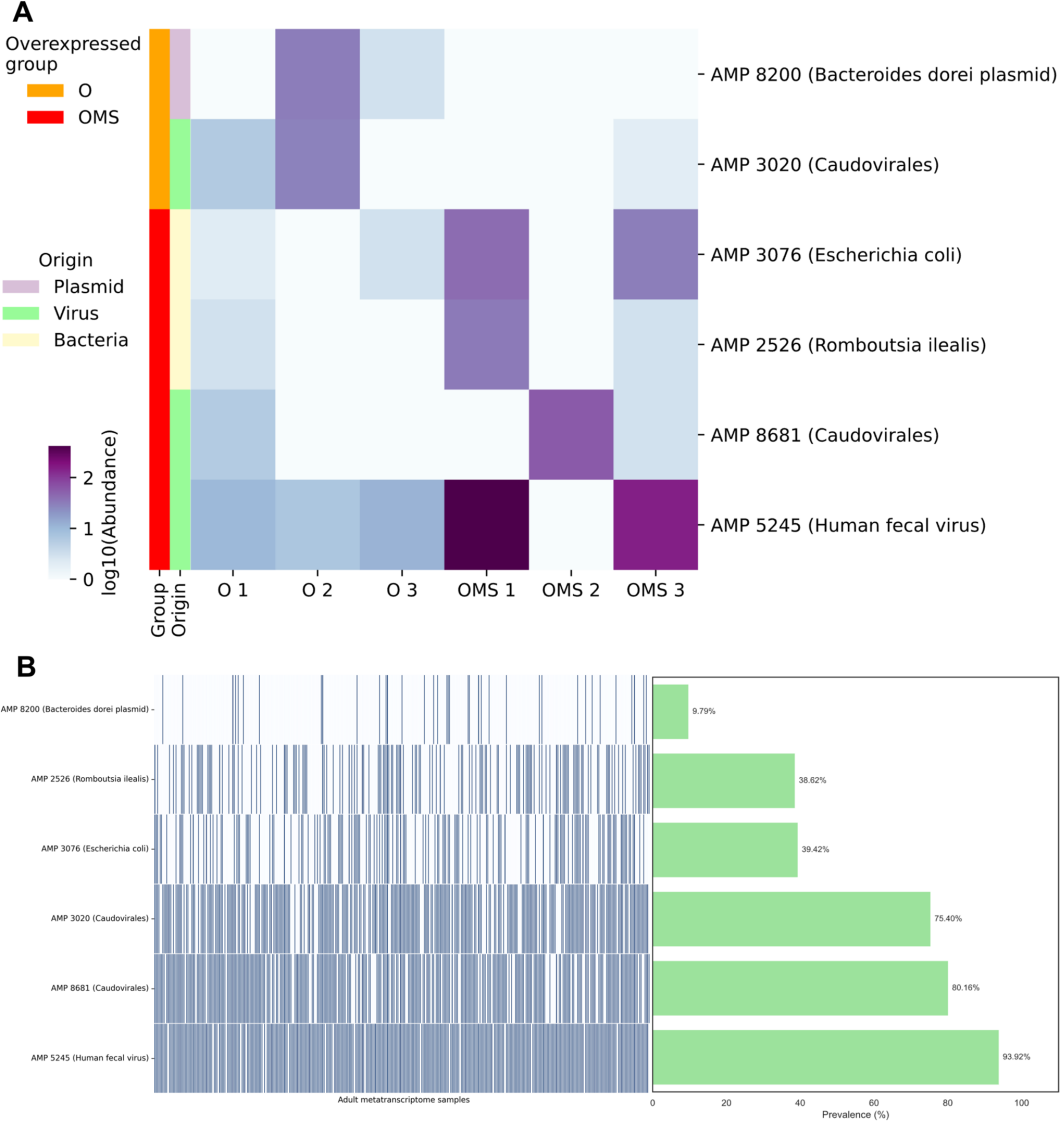
Overexpression of AMPs associated to obesity and MetS and their prevalence in external datasets. **A** Differential expression heatmap. Rows are AMPs; columns are samples. Row sidebars denote the overexpressed group (O = Obesity or OMS = obesity with MetS), predicted genomic origin (chromosome, plasmid or virus), and the lowest-level taxonomic assignment. **B** External prevalence. Presence of differentially expressed AMPs across 372 independent human gut metatranscriptomes (BioProject PRJNA354235)

We compared the expression of the 51 AMPs between the O and OMS groups (*n* = 3 per group). Using DESeq2 with thresholds of fold change > 2 and p-value < 0.05, 6 of 51 AMPs were differentially expressed: two up-regulated in O and four in OMS (Fig. 2A). The taxonomic assignment of these six AMPs revealed a mixture of phage-derived, plasmid-associated, and chromosomally encoded bacterial peptides (Fig. 2A; Supplementary Table 4), suggesting multiple genomic contexts for AMP expression in the gut. The differential expression of these AMPs in O vs. OMS suggests that AMPs dynamics may play a role in the dysbiosis associated with the disease.

To assess biological generalizability, we assessed whether these six AMPs are widely distributed across diverse human gut communities or if they are specific to our cohort. Accordingly, we queried the presence of the six AMPs in an independent dataset of 372 fecal metatranscriptomes from the U.S.-based Health Professionals Follow-Up Study (HPFS; Bioproject PRJNA354235) [37]. This cohort spans a wide range of BMI values, metabolic phenotypes, ages, and dietary backgrounds, thereby offering a robust framework for assessing AMP prevalence across heterogeneous populations. RNA-seq reads were mapped to the six AMP-encoding transcripts, and an AMP was considered detected at transcript per million (TPM) > 1. Notably, all six AMPs were observed in a substantial fraction of samples (prevalence 9.8–98.4%; mean 67.93%; median 80.16%; Fig. 2B; Supplementary Table 5), indicating widespread transcription across an independent population. These results point to recurrent AMP expression in the human gut and motivate future work to test their roles in interbacterial antagonism, community structure, and host–microbe signaling. 

### Chromosomal and Plasmid-Encoded AMPs Exhibit a Conserved Gut AMP Repertoire Across Taxa

Among the six differentially expressed AMPs, two had close protein homologs on bacterial chromosomes with phylogenetically diverse hosts. Specifically, AMP 3076 was identical (100% amino acid sequence identity) to homologs in *Escherichia coli* (Supplementary Fig.  3A and B); AMP 2526 shared 93.75% identity with four *Romboutsia* homologs (Supplementary Fig.  3C and D). The genomic context analyses revealed conserved local synteny around the two AMP surrounding genes, suggesting they are part of conserved genomic modules (Supplementary Fig. 4). The conservation in amino acid sequence and genomic architecture of these AMPs suggests strong purifying selection.

**Fig. 3:**
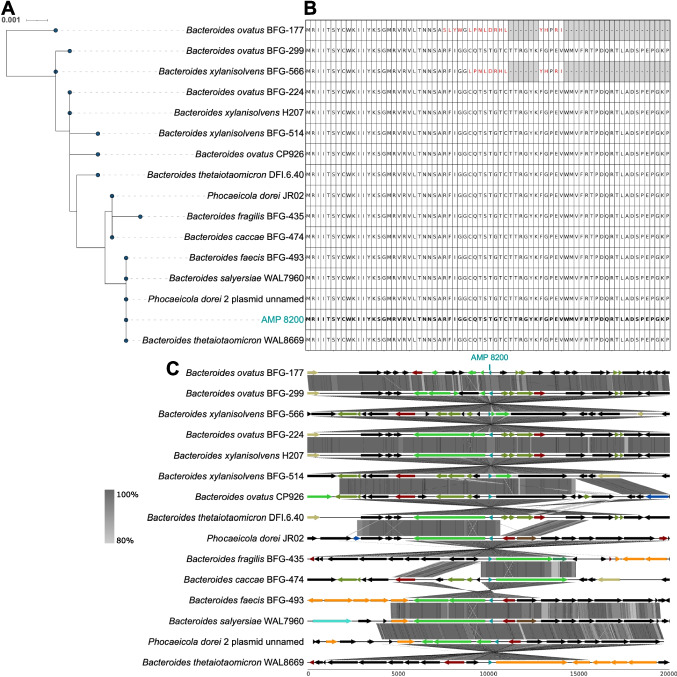
Molecular phylogeny (transcript), sequence conservation (protein), and conserved synteny (protein) of plasmid-derived AMP 8200. **A** Molecular phylogeny. Neighbor-Joining tree of AMP 8200 homologs. **B** Sequence conservation. Multiple alignment of AMP 8200 homologs; red color indicates the amino acid differences compared to the AMP sequence. **C** Conserved synteny. Gene neighborhoods flanking the AMP 8200 locus across representative genomes. Arrows indicate gene orientation; intergenic distances are to scale. Genes are colored by COG functional category; mobile-element genes are highlighted. The colors for functional categories were as follows: translation, ribosomal structure and biogenesis (blue); RNA processing and modification (forest green); transcription (dark red); replication, recombination and repair (orange); chromatin structure and dynamics (purple); cell cycle control, cell division, and chromosome partitioning (golden yellow); nuclear structure (steel blue); defense mechanisms (firebrick red); signal transduction mechanisms (lime green); cell wall/membrane/envelope biogenesis (dark brown); cell motility (cyan); cytoskeleton (royal blue); extracellular structures (slate gray); intracellular trafficking, secretion, and vesicular transport (magenta); posttranslational modification, protein turnover, and chaperones (dark orange); mobilome elements such as prophages and transposons (olive green); energy production and conversion (tomato red); carbohydrate transport and metabolism (turquoise); amino acid transport and metabolism (deep pink); nucleotide transport and metabolism (violet); coenzyme transport and metabolism (sea green); lipid transport and metabolism (khaki); inorganic ion transport and metabolism (cobalt blue); secondary metabolite biosynthesis, transport, and catabolism (indian red); general function prediction only (dark gray); and unknown function (black)

Among chromosomally encoded AMPs, AMP 8200 was notable for its dual genomic occurrence. BLASTP searches identified 15 highly similar protein homologs across *Bacteroides* and *Phocaeicola*, including one copy on a *Phocaeicola dorei* plasmid (Fig. 3A and B). The peptide sequence was 100% conserved in 13 strains, indicating strong purifying selection on this AMP. Additionally, synteny was preserved across host genomes, suggesting a conserved functional genomic region (Fig. 3C). The presence of AMP 8200 in both chromosome and plasmid suggests a potential horizontal transfer event. Thus, we examined whether the 85 kb plasmid carrying the AMP gene was integrated into the chromosome of *P. dorei* strain JR01. Whole-genome alignment indicated that only a 13 kb plasmid fragment (containing the AMP gene) was integrated into the chromosome, rather than the entire plasmid (Supplementary Fig.  5A). To distinguish between plasmid and chromosome origin of transcripts, we re-mapped the RNA-seq reads to the shared 13 kb region. Alignments to the plasmid sequence showed perfect matches, whereas alignments to the chromosomal counterpart contained 17 nucleotide mismatches. This suggests that transcripts mainly originate from the plasmid. Interestingly, when the RNA-seq reads were mapped to the plasmid, we found extensive transcriptional activity across the entire plasmid (95.5% plasmid coverage with mean depth 61.7X) (Supplementary Fig. 5B). These results underscores that mobile genetic elements in the gut can be highly transcribed and may disseminate antimicrobial functions within the community.

### AMPs Encoded in Bioactive Phages Suggest the Presence of Phage-Host Dynamics

**Fig. 4:**
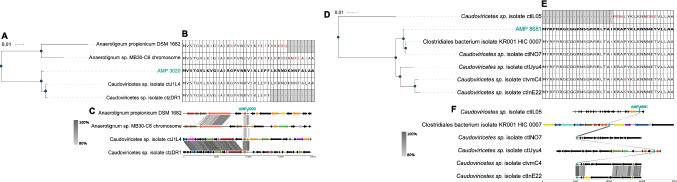
Molecular phylogeny (transcript), sequence conservation (protein), and conserved synteny (protein) of phage-derived AMP 3020 (**A**, **B** and **C**) and AMP 8681 (**D**, **E** and **F**). Molecular phylogeny. Neighbor-Joining tree of AMP 3020 (**A**) and AMP 8681 (**D**) homologs. Sequence conservation. Multiple alignment of AMP 3020 (**B**) and AMP 8681 (**E**) homologs; red color indicates the amino acid differences compared to the AMP sequence. Conserved synteny. Gene neighborhoods flanking the AMP 3020 (**C**) and AMP 8681 (**F**) locus across representative genomes. Arrows indicate gene orientation; intergenic distances are to scale. Genes are colored by COG functional category; mobile-element genes are highlighted. The colors were represented as detailed in the legend of Fig. 3

Three AMPs (AMP 3020; AMP 8681, and AMP 5245) show high sequence similarity to proteins from tailed bacteriophages (Fig. 4), pointing to active phage involvement. For AMP 3020 BLASTP analysis demonstrated a perfect match (100% identity) to proteins from Caudoviricetes phages ctJ1L4 and ctzDR1, and 70.9–74.4% identity to two homologs from *Anaerotignum* strains (Fig. 4A and B). Despite these high sequence similarities, AMP 3020 displayed poor synteny conservation with their surrounding genes in both phages and bacterial genomes (Fig. 4C).

The identical sequence between AMP 3020 and the homolog in phage ctJ1L4 suggests that their overexpression originated from this phage and not from *Anaerotignum* bacteria. To determine the source of expression, we mapped viral-like particle (VLP) DNA-seq reads from the same samples previously published by our laboratory [9] to ctJ1L4 phage genome. After mapping*,* we found that 70.46% of the genome was covered by virome derived reads, providing strong evidence for the physical presence of this phage as a viral particle, because DNA was isolated from purified VLPs (Supplementary Fig.  6A). Furthermore, the RNA-seq mapping covered 14.06% of the phage genome, indicating transcription of several phage genes (Supplementary Fig. 6B). Together, these data support ctJ1L4 as the likely source of AMP 3020 transcripts rather than *Anaerotignum*.

Given that AMP 3020 also shared 74.36% sequence identity with a protein from *Anaerotignum* sp. MB30-C6 genome, we investigated if the ctJ1L4 phage was integrated into the bacterial chromosome. Only a 372 nucleotides segment of the 32.5 kb ctJ1L4 genome aligned to *Anaerotignum* sp. MB30-C6, corresponding to the AMP 3020 locus (Supplementary Fig.  6C). This suggest that the phage genome was not integrated into the bacterial chromosome. Prophage prediction analysis in the *Anaerotignum* MB30-C6 genome did not identify prophages containing the AMP, further suggesting the bacterial copy is not part of a prophage.

The AMP 8681 was identical (100% sequence identity) to proteins from two Caudoviricetes phages (ctlN07 and ctUyu4) and Clostridiales bacterium KR001 hic 0007, and shows 96.97% identity to proteins from phages ctvmC4 and ctlnE22 (Fig. 4D and E). Despite this high sequence conservation between proteins, synteny around the locus was weakly conserved in both phage and bacterial genomes (Fig. 4F). Given that AMP 8681 had identical homolog proteins in both bacteria and phages, we investigated which of these two genomes could be the potential source of AMP overexpression. Mapping virome-derived reads to ctlN07 phage showed limited coverage (7.18%) (Supplementary Fig.  7A), whereas RNA-seq mapping covered 12.82% of the genome at 52.65X mean depth, indicating transcription despite low DNA abundance(Supplementary Fig. 7B). We evaluated possible phage integration by comparing the 50 kb *ctlN07* genome to the Clostridiales bacterium KR001 hic 0007 genome, and found only a 3-kb shared region (98.10% identity) encompassing the AMP locus, indicating no phage integration in the bacterial genome (Supplementary Fig.  7C). Additionally, prophage prediction in the bacterial genome did not identify ctlN07 or other prophages carrying the AMP, suggesting a non-viral origin of this AMP in the bacterial genome. Thus, AMP 8681 overexpression could be originated from either the phage or the bacterium additional functional assays are needed to resolve the primary source.

**Fig. 5:**
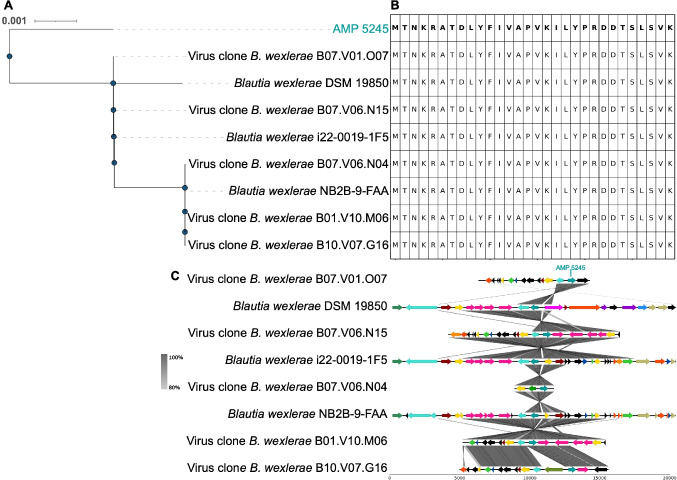
Molecular phylogeny (transcript), sequence conservation (protein), and conserved synteny (protein) of phage-derived AMP 5245. **A** Molecular phylogeny. Neighbor-Joining tree of AMP 5245 homologs. **B** Sequence conservation. Multiple alignment of AMP 5245 homologs; red color indicates the amino acid differences compared to the AMP sequence. **C** Conserved synteny. Gene neighborhoods flanking the AMP 5245 locus across representative genomes. Arrows indicate gene orientation; intergenic distances are to scale. Genes are colored by COG functional category; mobile-element genes are highlighted. The colors were represented as detailed in the legend of Fig. 3

The AMP 5245 shows 100% identity to multiple phage proteins and to homologs in *Blautia wexlerae* (Fig. 5A and B), with two conserved genomic architectures spanning phages and bacterial contexts (Fig. 5C). We investigated the source of the observed AMP overexpression. VLP DNA-Seq reads covered 88.7% of the cognate phage genome, and RNA-seq reads covered 86.7% at a mean depth 1,777X, indicating an strong transcription of the majority of phage genes (Supplementary Fig.  8A and B). Next, we analyzed if phage genome was integrated into the bacterial genome, and the alignment of the 8-kb human fecal virus clone to *B. wexlerae* DSM 19850 revealed a 3-kb region (98.35% identity), containing the AMP locus (Supplementary Fig.  8C), suggesting a partial integration of phage (39.53% of the phage genome) into the bacterial genome. However, prophage prediction in *B. wexlerae* did not detect any prophage elements matching the human fecal virus clone, arguing against integration. Additionally, prophage prediction did not detect any other prophages containing the AMP. The high percentage of phage genome coverage with VLP DNA and RNA reads therefore implicates the phage as the principal source of AMP 5245 transcripts. Collectively, mapping and synteny analyses support phage origin and active transcription for AMP 3020 and AMP 5245, while AMP 8681 remains ambiguous, highlighting dynamic phage–host interactions and potential bidirectional exchange of AMP genes.

### Correlation Analysis links AMPs Expression with Bacterial Taxa

**Fig. 6:**
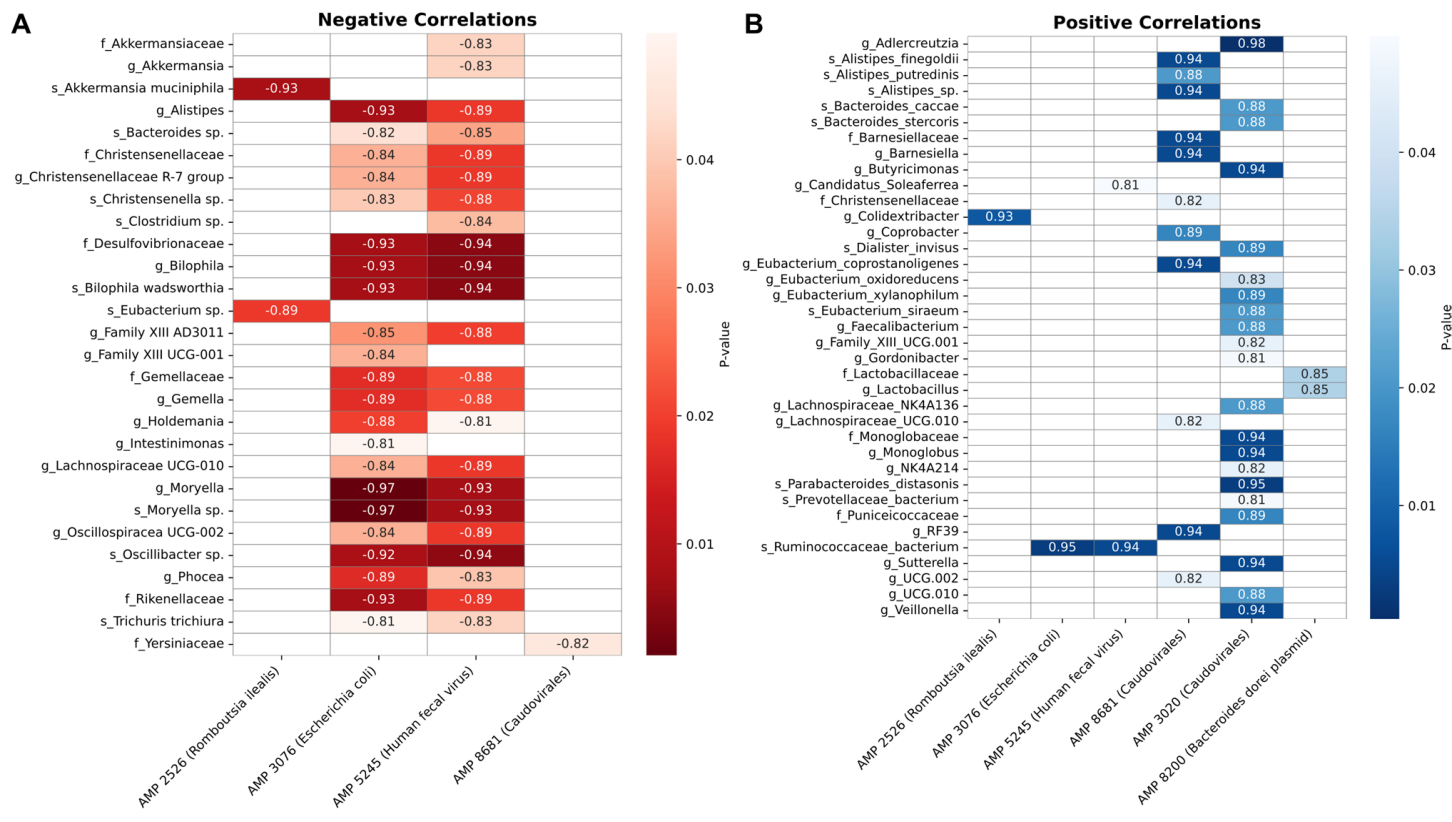
Heat-map of correlation analysis between bacterial taxa (16S) and AMP expression. **A** Negative correlations. **B** Positive correlations. Columns are AMPs; rows are taxa. Color indicates correlation strength (p-value). Value inner box indicates the correlation value. Only correlations passing significance were displayed (*p* < 0.05)

To explore associations between the six overexpressed AMPs and gut microbiota, we correlated the AMP expression with 16S rRNA profiles from the same samples (BioProject PRJNA600247). We observed significant negative correlations in which higher AMP expression coincided with lower taxon abundance (Fig. 6A). Notable inverse correlations included: AMP 5865 with *Anaeroplasma*, *Clostridium*, and *Bacteroides* spp.; AMP 5245 with > 20 taxa, prominently *Akkermansia*, Christensenellaceae, *Moryella*, and *Oscillibacter*; AMP 3076 with Christensenellaceae, *Bilophila*, and multiple Lachnospiraceae lineages; AMP 3096 with Desulfovibrionaceae, *Bilophila wadsworthia*, *Oscillibacter*, and *A. muciniphila*; and AMPs 2526 and AMP 2198 with *A. muciniphila* and *Eubacterium* spp.

Beyond inverse associations, we also identified statistically significant positive correlations between the expression of several AMPs and specific bacterial taxa (Fig. 6B). These likely reflect ecological compatibility, co-expression via shared mobile elements, or shared niche responses. Notable positive correlations included: AMPs 2198 and 2526 with *Colidextribacter*; AMPs 3076, 5245 and 3096 with Candidatus Soleaferrea; AMP 8681 with several *Alistipes *spp. and > 11 taxa; and AMP 3020 with > 21 taxa, including *Adlercreutzia*, *Butyricimonas*, *Dialister invisus*, several *Eubacterium *spp., *Gordonibacter*, *Monoglobus*, Prevotellaceae bacterium, and *Sutterella*.

Taken together, the inverse correlations (often involving gut health–associated taxa (Table 1)) and the positive correlations (frequently implicating obesity-linked lineages (Table 1)) suggest a model in which AMP expression contributes to microbiome restructuring in obesity/MetS, directly constraining beneficial taxa and indirectly favoring obesity-associated taxa via competitor suppression (Fig. 8). However, these are observational, non-causal associations; given the modest sample size, we consider them exploratory and hypothesis-generating, meriting confirmation in larger cohorts and targeted mechanistic studies.

**Table 1:** Correlations between gut-expressed antimicrobial peptides (AMPs) and bacterial taxa

AMP (ID)	Correlation (direction)	Correlated taxa (Family/Genus/species)	Healthy or Obesity -related association	References
AMP 2526	↓ Negative	*A. muciniphila*	Healthy linked	[52, 61]
AMP 2526	↓ Negative	*Eubacterium spp.*	Mixed	[63, 64]
AMP 3076 and AMP 5245	↓ Negative	*Bacteroides, Oscillibacter, Alistipes, Holdemia, Christensenella spp, Christensenellaceae R-7 group, Lachnospiraceae UCG-010*	Healthy linked	[54–59, 65–68]
AMP 3076	↓ Negative	*Intestimonas*	Healthy linked	[69]
AMP 5245	↓ Negative	*Clostridium spp.*	Mixed	[70, 71]
AMP 5245	↓ Negative	*Akkermansia*	Healthy linked	[52, 72]
AMP 8681	↓ Negative	*Yersiniaceae*	Obesity linked	[73]
AMP 2526	↑ Positive	*Colidextribacter*	Obesity linked	[53, 74]
AMP 3076 and AMP 5245	↑ Positive	*Ruminococcaceae bacterium*	Obesity linked	[60]
AMP 5245	↑ Positive	*Candidatus Soleaferrea*	Mixed	[61]
AMP 8681	↑ Positive	*Alistipes spp., Barnesiella, Coprobacter, Eubacterium coprostanoligenes, Lachnospiraceae UCG-010, RF39*	Healthy linked	[54–59, 65–68]
AMP 3020	↑ Positive	*Adlercreutzia, Dialister invisus, Eubacterium oxidoreducens, Monoglobus, Family XIII UCG-001, Sutterella, Veillonella*	Obesity linked	[75–81]
AMP 3020	↑ Positive	*Bacteroides caccae, Butyricimonas, Prevotellaceae bacterium, Veillonella*	Mixed	[82–84]
AMP 3020	↑ Positive	*Bacteroides stercoris, Eubacterium coprostanoligenes, Eubacterium xylanophilum, Eubacterium siraeum, Faecalibacterium, Parabacteroides distasonis*	Healthy linked	[63, 66, 85–88]
AMP 8200	↑ Positive	*Lactobacillus*	Healthy linked	[89]

The table lists AMP identifiers (AMP ID), the direction of the association (Direction; ↑ positive, ↓ negative) between AMP expression and the relative abundance of each taxon across samples, the most specific taxonomic label available (Correlated taxa; Family/Genus/species), and a literature-based summary of whether each taxon is typically linked to healthier or obesity-related states (Healthy or Obesity-related association). Mixed indicates heterogeneous or context-dependent evidence acrossstudies. Joint entries (e.g., “AMP 3076 and AMP 5245”) indicate that both AMPs share the same direction of association with the listed taxon

### Experimental Validation of Phage-Encoded AMP 3020: Broad-Spectrum Antibacterial Activity without Detectable T-Cell Toxicity

**Fig. 7:**
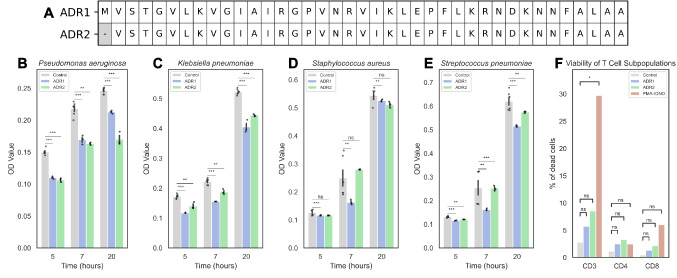
In-vitro characterization of phage-encoded AMP 3020. **A** Amino acid sequences of AMP 3020 (ADR1) and its variant (ADR2). (B-E) Time-course antimicrobial activity against **B**) *Pseudomonas aeruginosa*, **C**) *Klebsiella pneumoniae*, **D**) *Staphylococcus aureus*, and **E**) *Streptococcus pneumoniae*. **F**) Viability of T cell subpopulations after 24 h exposure to 20 µg of ADR1 or ADR2. Bars show mean ± standard deviation. Statistical significance was assessed using two-sided t-tests: ns = not significant, * = *p* < 0.05, ** = *p* < 0.01, *** = *p* < 0.001, **** = *p* < 0.0001

To test our in-silico predictions, we selected the AMP 3020 for experimental validation based on its confirmed phage origin. We synthesized two variants (Fig. 7A): ADR1 which retains the initiator methionine, and ADR2, which lacks this residue and begins with valine. ADR2 starts with valine, as it has been reported that some peptides undergo post-translational processing when the second residue of a nascent peptide is a short one; here, the second residue was valine [38]. This single amino acid difference also allowed us to assess whether minimal N-terminal changes modulate activity. Both peptides significantly inhibited the growth of Gram-negative bacteria (*Pseudomonas aeruginosa* and *Klebsiella pneumoniae*) and Gram-positive bacteria (*Staphylococcus aureus* and *Streptococcus pneumoniae*) relative to controls (Fig. 7B–E). ADR1 showed greater potency against *P. aeruginosa*, whereas ADR2 was more effictive against *K. pneumoniae*. Both variants exhibited antibacterial activity against *S. pneumoniae*, with only ADR1 inhibited *S. aureus*. Notably, neither peptide impaired primary human T-lymphocyte viability (Fig. 7F), with similar cell death frequencies (0.7–14.4%) to those of untreated controls (Fig. 7F). The absolute cell counts confirmed no significant loss of viable T cells, in contrast to the cytotoxic positive control (PMA/ionomycin) (Supplementary Figs. 9 and 10). Overall, ADR1 and ADR2 exhibited antibacterial activity with no detectable T-cell toxicity under our assay conditions. The absence of the initiator methionine in ADR2 may subtly alter peptide folding or charge distribution and thereby tune target specificity, a hypothesis that merits future mechanistic testing.

## Discussion

This study provides a framework for discovering and characterizing AMPs in the human gut microbiome using metatranscriptomics and viromics, representing a promising frontier to understand their role in host-microbiota interactions [10]. Using our methodology described in Fig. 1 we identified 51 high-confidence AMP candidates from 1,095 expressed small open reading frames (smORFs). The limited overlap among the results of the different AMP prediction tools highlights the methodological variability in AMP discovery. Notably, many expressed AMPs were linked to *Faecalibacterium prausnitzii*, a known gut commensal bacterium. Additionally, the identification of AMP-encoding transcripts from plasmids and phages expands the potential origins of AMPs beyond traditional chromosomal sources. One of the key smORF quality criteria proposed by Sberro et al*.* is to reduce false positives by retaining only smORFs that contain both start and stop codons [19]. However, metatranscriptomic assemblies often yield short contigs representing partial transcripts. To address this, we leveraged homologs transcripts and their predicted proteins to complete the coding sequences of our AMPs. This step was critical for eliminating protein truncation artifacts. Notably, our AMPs aligned to their reference peptides with a mean coverage of 92% and a mean sequence identity of 97%, indicating that only small N- or C-terminal fragments were missing in the RNAseq.

**Fig. 8:**
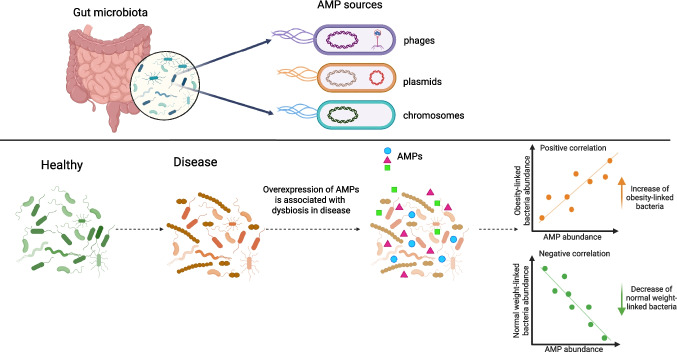
Gut-expressed antimicrobial peptides (AMPs) originated from bacterial chromosomes, plasmids, and phages. AMP over-expression negatively correlates with healthy-associated taxa and positively correlates with obesity-linked taxa. The microbiota dysbiosis observed in obesity and Metabolic syndrome can be associated with AMP overexpression. Icons indicate AMP source; arrows show correlation direction.  Created in BioRender: https://BioRender.com/w7dlcld

The intestinal epithelial interface contains AMPs of both host and microbial origin, which play a crucial role in shaping the surrounding microbiota [39]. Consistent with emerging evidence that bioactive peptides can remodels gut microbiota [40], our data suggests that AMPs may contribute to the microbiota alterations observed in obesity and metabolic syndrome [8]. Notably, the two phage-derived AMP 3020 variants (ADR1 and ADR2) displayed selective antibacterial activity while remaining non-cytotoxic to primary human T-cell subsets (CD3 +, CD4 +, and CD8 +). This profile aligns them with host defense peptides (HDPs), a broader class that includes bacteriophage-encoded peptides capable of modulating inter-microbial competition and host-microbiota interactions [41, 42]. The absence of T cell cytotoxicity emphasizes the immunological neutrality of these peptides in vitro, suggesting some phage encoded AMPs produced within the gut can reshaped bacterial communities without directly harming host. These findings advance our understanding of virome-microbe crosstalk and suggest that phage-derived AMPs may influence ecosystem composition while preserving host immune balance [43, 44]. Moreover, the obesity-associated upregulation of several AMPs and their negative correlations with taxa linked to metabolic health (Table 1) is consistent with AMP-mediated pressures, suggesting their potential role favoring dysbiosis linked to obesity and MetS (Fig. 8). While these associations are not evidence of causality, they motivate mechanistic studies (e.g., gnotobiotic models and in situ peptide perturbations) to test whether phage-encoded AMPs can drive microbiota shifts relevant to metabolic disease.Differential expression analysis comparing obesity with obesity and metabolic syndrome identified six AMPs that were significantly overexpressed, originating from chromosomal, plasmid, and phage genomic contexts. This supports the idea that the host metabolic status influences microbial gene expression for AMP production. Importantly, all sixAMPs were also detected in 372 samples from an independent gut metatranscriptome dataset, suggesting their commonality and ecological significance in the human gut microbiome, rather than being unique to our cohort. Their widespread prevalence argues for ecological relevance and nominates them as candidate biomarkers of microbiome perturbations associated with metabolic disease (Fig. 8).

We identified three AMPs with close homologs in phages, an underexplored reservoir of antimicrobial compounds. AMP 3020 shared 98% identity with a protein from the Caudoviricetes phage ctJ1L4. DNA VLP-virome read mapping suggested the presence of ctJ1L4 virions and metatranscriptome read mapping shows an active transcription of multiple phage genes, including the AMP. Synthetic AMP 3020 inhibited both Gram-positive and Gram-negative bacteria, consistent with broad-spectrum activity. Although related proteins exist in two bacterial genomes, we found no evidence of ctJ1L4 integration or prophages carrying the AMP gene, suggesting that in vivo AMP trasncription stemmed primarily from lytic phage activity, which was suggested by DNA VLP-virome read mapping. Removal of the N-terminal Methionine altered antibacterial activity, reflecting the evolutionary adaptability of phage-derived peptides. Methionine is essential for stabilizing protein structures and may also act as a regulatory switch through reversible redox reactions [45]. Overall, these findings expand the functional repertoire of gut phages, particularly those within the dominant Caudovirales lineage found in the human gut virome [9] and reinforce phages as a rich, underrecognized source of AMPs capable of reshaping microbioal communities (Fig. 8). Future mechanistic studies in phage–host systems will be essential to establish causality and therapeutic potential.

AMPs 8681 and 5245 show 100% amino acid identity between their phage- and bacterium-encoded homologs. DNA VLP virome read mapping suggests the phage presence. Additionally, metatranscriptomic data showed active transcription of several phage genes, suggesting that phages substantially contributed of AMP transcripts in vivo, although bacterial loci may also play a role in the transcription. Prophage scans show that both chromosomal AMP loci were located outside predicted prophage regions, suggesting that the integration of both AMPs into the bacterial genomes was not related to a prophage. Interestingly, the expression of AMP 5245 negatively correlated with Moryella and Eubacterium species, known butyrate producers with anti-inflammatory effects, suggesting that this AMP may antagonize taxa linked to intestinal health [46, 47]. We also detected a plasmid-encoded homolog protein of AMP 8200, a plasmid described in *Phocaeicola dorei* [48]. The peptide exhibits 100% sequence identity and conserved synteny across additional *Phocaeicola* species. RNA-seq read mapping revealed robust transcription across the entire plasmid, suggesting the plasmid as the primary source of AMP transcripts, reinforcing the concept that plasmids serve as reservoirs of AMPs in the gut microbiome [49]. Together, these observations highlight the potential for plasmids and phages to spread competitive traits such as AMPs via horizontal transfer.

Additional AMPs 3076 and 2526, shared homolog proteins across diverse gut bacterial commensals, including *Escherichia coli* and *Romboutsia* spp., suggesting their widespread distribution in the intestinal microbiota. These AMPs demonstrated high sequence and synteny conservation across bacterial genomes. Expression of these AMPs negatively correlated with microbial groups associated with metabolic health, such as *Akkermansia muciniphila and Christensenellaceae,* suggesting a potential link with microbial dysbiosis observed in obesity and metabolic syndrome [50, 51].

Given our modest sample size, the correlations between AMPs and disease-associated taxa should be viewed as exploratory. Interestingly, AMP 2526 (overexpressed in OMS) was negatively correlated with *A. muciniphila*, taxa frequently linked to metabolic health (Table 1) [52]. By contrast, AMP 2526 correlated positively with Colidextribacter, a genus increased in obese mice and associated with higher body/liver weight and circulating lipids (Table 1) [53]. Overall, the correlation pattern links AMP 2526 to an obesogenic microbiome: it is negatively associated with taxa typically tied to metabolic health and positively associated with obesogenic taxa, suggesting AMP 2526 may mark, or contribute to, dysbiosis and adverse metabolic status in OMS, but these are associations only. Validation in larger, independent cohorts and mechanistic models is required before inferring causality. Another example was the AMPs 3076 and 5245 negatively correlated with several healthy linked taxa, such as *Bacteroides* and *Oscillibacter* (Table 1). Furthermore, these AMPs also were negatively *Alistipes* which was enriched in normal-weight individuals compared to subjects with obesity [54, 55]; *Holdemania* which is is more abundant in normal-weight children [56]; and Christensenella spp., that reduce diet-induced weight gain, dyslipidemia, and hepatic steatosis in mice [57]. These AMPs also were negatively correlated with Christensenellaceae R-7 group, Lachnospiraceae UCG-010, and Rikenellaceae which are linked to healthier metabolic profiles (Table 1) [58, 59]. This suggests the possibility that both AMPs may constrain these beneficial taxa. Nonetheless, these observations require validation in larger, independent cohorts and experimental systems before inferring causality. Another example was the AMPs and 5245 negatively correlated with several healthy linked taxa, such as *Bacteroides* and *Oscillibacter* (Table 1). Furthermore, these AMPs also were negatively *Alistipes* which was enriched in normal-weight individuals compared to subjects with obesity [54, 55]; *Holdemania* which is is more abundant in normal-weight children [56]; and Christensenella spp., that reduce diet-induced weight gain, dyslipidemia, and hepatic steatosis in mice [57]. These AMPs also were negatively correlated with Christensenellaceae R-7 group, Lachnospiraceae UCG-010, and Rikenellaceae which are linked to healthier metabolic profiles (Table 1) [58, 59]. This suggest the possibility that both AMPs may constrain these beneficial taxa. Nonetheless, these observations require validation in larger, independent cohorts and experimental systems before inferring causality.

By contrast, AMPs 3076, and 5245, correlated positively with a Ruminococcaceae bacterium; a family shows context-dependent links to obesity, enriched in some cohorts, reduced or unchanged in others [60]. AMP 5245 also positively correlated with Candidatus, whose metabolic associations are mixed—reported as protective in some settings but linked to cardiometabolic risk in others [61]. Taken together, AMPs 3076 and 5245 align with depletion of taxa linked to leanness and some increases in taxa with mixed/obesogenic associations. Unlike the other overexpressed AMPs, AMP 8681 shows the opposite pattern: it associates negatively with taxa linked to adverse metabolic states and positively with taxa tied to metabolic health. Specifically, it correlates inversely with Yersiniaceae (often elevated in obesity [62] and positively with  *Alistipes* spp., *Barnesiella*, *Coprobacter*, *Eubacterium coprostanoligenes*, Lachnospiraceae UCG-010, and the RF39 clade—groups typically more abundant in lean or metabolically healthy cohorts, as early described. These are correlations only and likely reflect context-dependent shifts rather than direct AMP effects.

Overall, these patterns suggest potential coexistence or co-regulation, which may arise from: (i) ecological compatibility—AMP activity suppressing competitors and indirectly favoring non-target taxa; (ii) co-expression driven by mobile elements (plasmids/phages) carrying both AMP genes and host-maintenance functions; and/or (iii) shared responses to niche conditions (e.g., substrates, stress signals) that simultaneously elevate AMP transcription and the abundance of certain lineages. These positive co-occurrence patterns, together with the inverse correlations reported above, point to structured community responses to AMP expression—encompassing both potential constraint of sensitive taxa and concomitant expansion of compatible or co-regulated lineages. Given the modest sample size, we interpret these findings as exploratory and hypothesis-generating; they nominate specific AMP–taxon pairs for targeted validation in larger cohorts and mechanistic experiments.

Metatranscriptomic profiling provides a dynamic map of microbiome activity, revealing which genes are actively expressed under conditions such as obesity and metabolic syndrome. This approach differs from traditional metagenomics, which only lists gene presence. Our analysis identified actively expressed AMPs, helping differentiate between latent genetic potential and those that actively influence host microbiota and disease associations. Our findings highlight that gut-expressed AMPs were derived from diverse genomic sources, including bacteria, plasmids, and phages, indicating their significant ecological roles in gut microbial dynamics. This proof-of-concept study shows that metatranscriptomic data can uncover relevant, expressed AMPs implicated in the gut microbiome regulation. The differential expression of these AMPs associated with disease, the negative and positive correlations with microbial taxa, along with their antimicrobial properties that do not affect host immunity points to their importance in shaping the gut microbiota. Moreover, the presence of mobile genetic elements, such as plasmids and phages, reinforces the need to rethink the role of phages in gut ecology, acting not only as predators but also as regulators. While our study was exploratory, it establishes metatranscriptomics and viromics as a practical framework to uncover relevant, expressed AMPs implicated in microbiome regulation (Fig. 8). This work sets the stage for future studies on AMPs and their therapeutic potential in microbiome-targeted treatments in obesity and MetS-associated dysbiosis.

## Supplementary Information

Below is the link to the electronic supplementary material.

Supplementary file1 (PDF 2.77 KB)

Supplementary file1 (PDF 37.2 KB)

## Data Availability

The data that support the findings of this study are openly available in NCBI GEO repository with accession number GSE143207 (https://www.ncbi.nlm.nih.gov/geo/query/acc.cgi?acc = GSE143207) and the NCBI BioProject: PRJNA600247 (https://www.ncbi.nlm.nih.gov/bioproject/?term = PRJNA600247) and PRJNA646512 (https://www.ncbi.nlm.nih.gov/bioproject/?term = PRJNA646512). All the code used for this project was deposited in this GitHub repository: (https:/github.com/LuiguiGallardo/amps_microbiome_2025). Requests for additional material should be made to the corresponding author.
