# Supplementary material for: Bioactive Plasmid- and Phage-Encoded Antimicrobial Peptides (AMPs) in the Human Gut: A Metatranscriptome–Virome Profiling Reveals Exploratory Links to Metabolic Human Diseases
Source: Microb Ecol. 2025 Nov 28;89:15. doi: 10.1007/s00248-025-02620-2 (PMC12775044; doi:10.1007/s00248-025-02620-2)
Supplement: Supplementary file 1 [file 248_2025_2620_MOESM1_ESM.pdf]

Supplementary figures

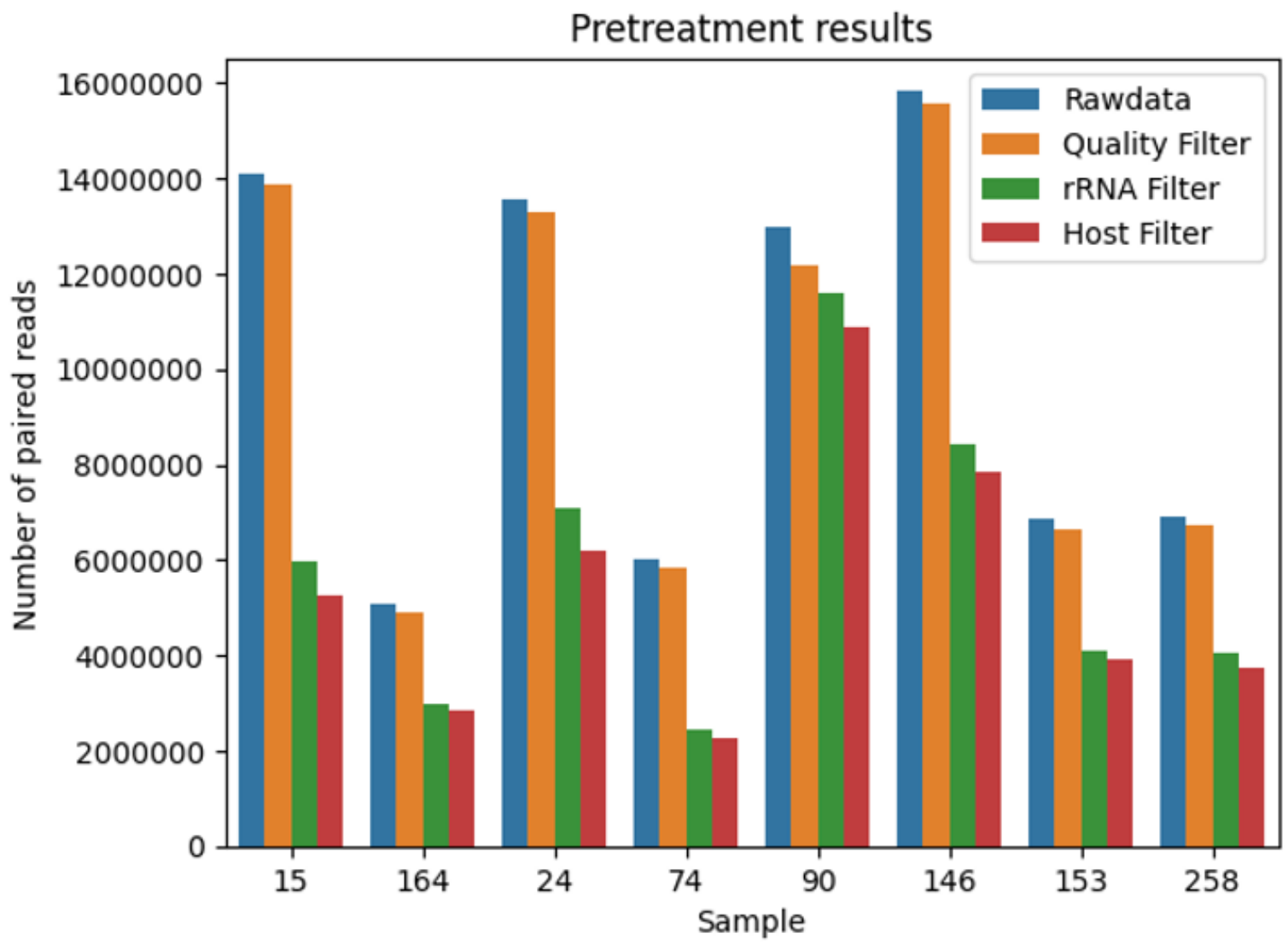

Supplementary Figure 1: Number of reads per sample for each pre-treatment step.

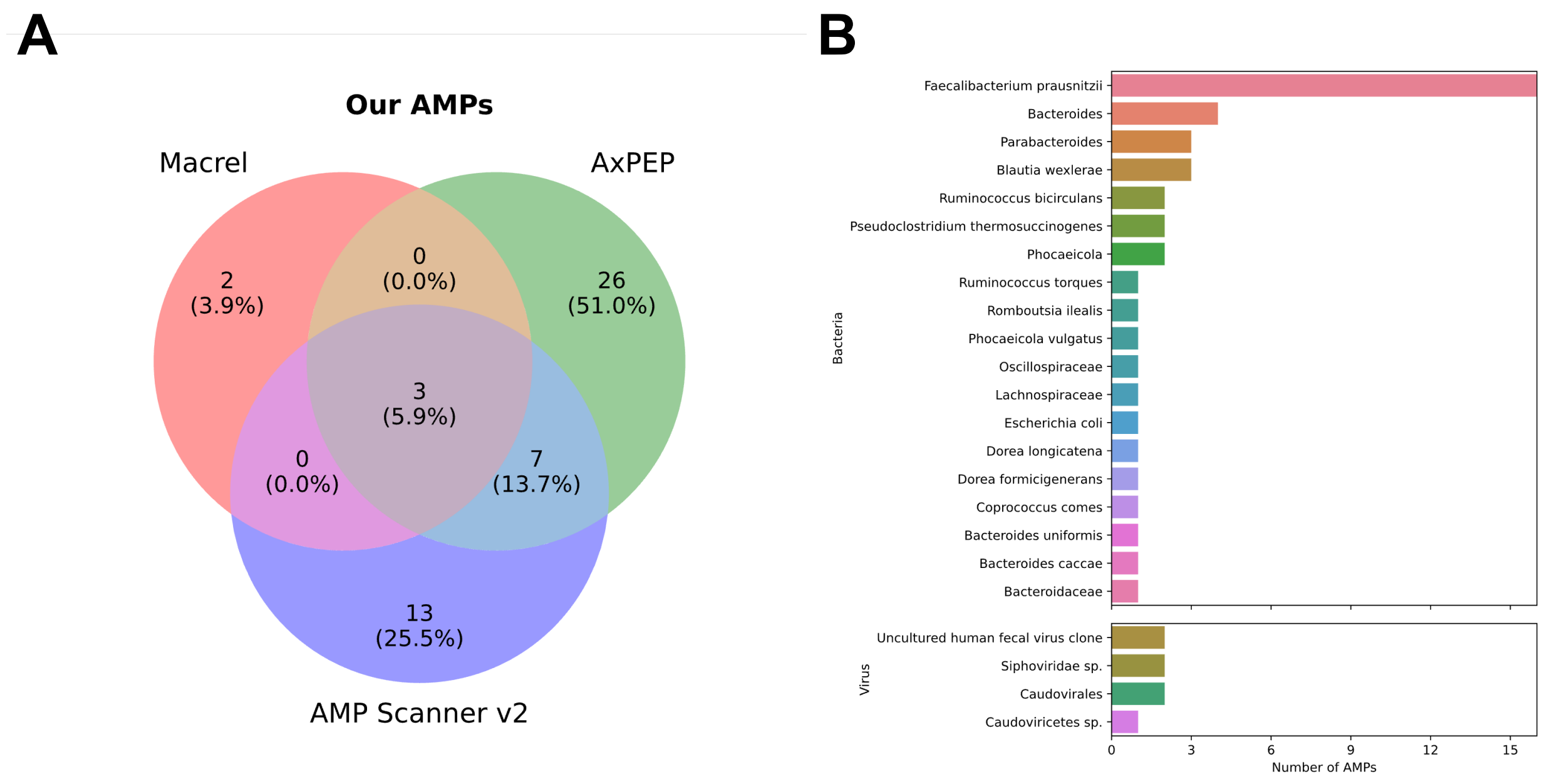

Supplementary Figure 2: A) Venn diagram illustrating the overlap of AMPs predicted using three different tools: Macrel, AMP Scanner v2, and AxPEP, from our metatranscriptome data. B) Identification and Taxonomic Origin of the 51 High Confidence AMPs, as determined by BLASTN and MEGAN6 analysis of the AMP-encoding sequences.

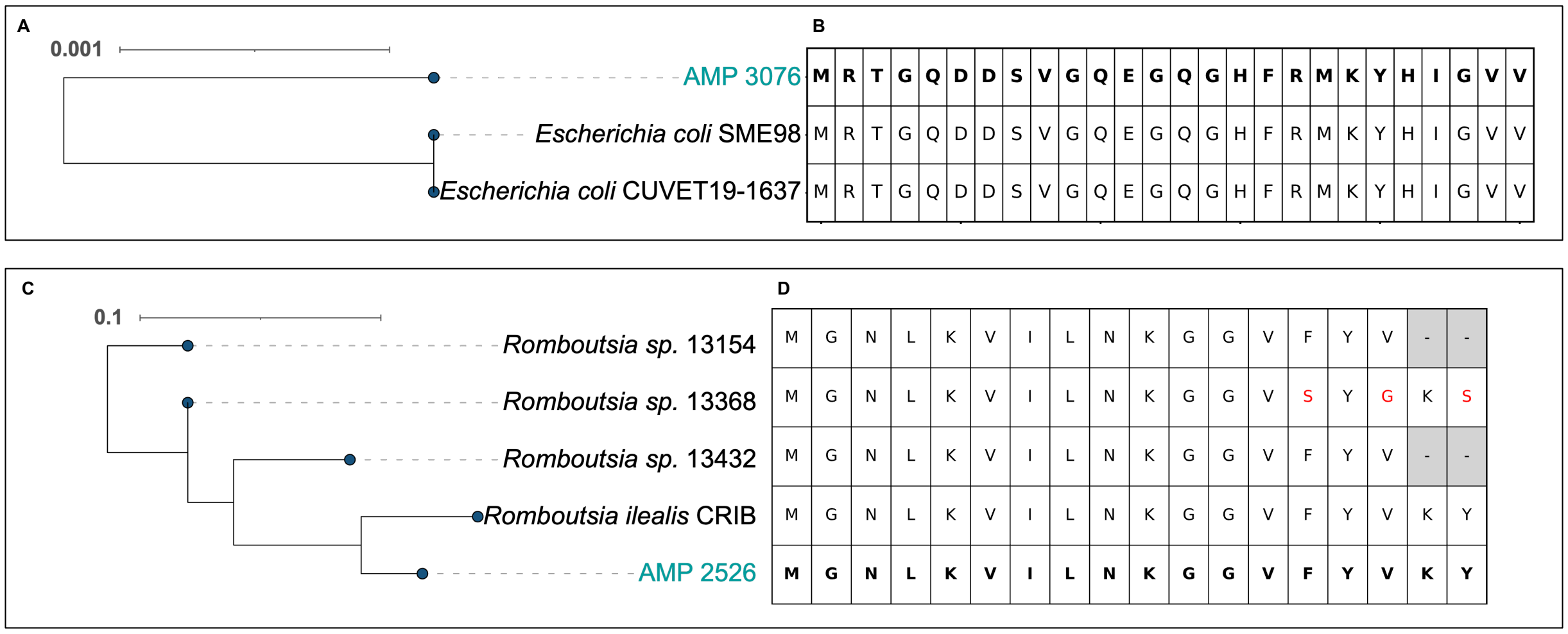

Supplementary Figure 3: Phylogenetic analysis of putative AMPs encoded by bacteria. A) AMP 3076 and B) AMP 2526

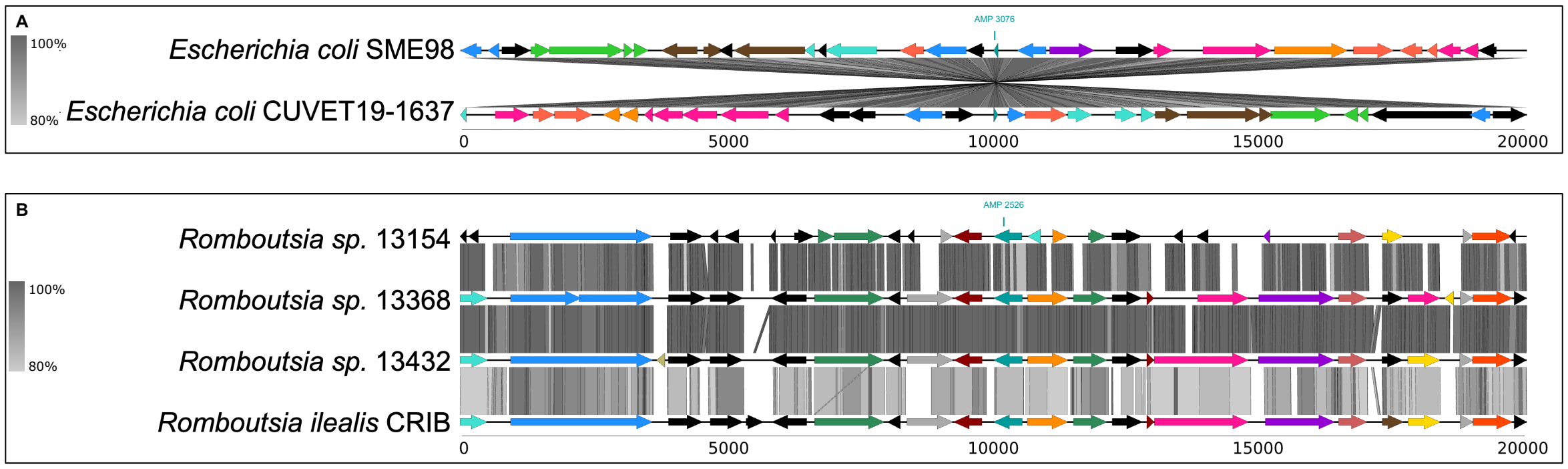

Supplementary Figure 4: Phylogenetic analysis of putative AMPs encoded by bacteria. A) AMP 3076 and B) AMP 2526.

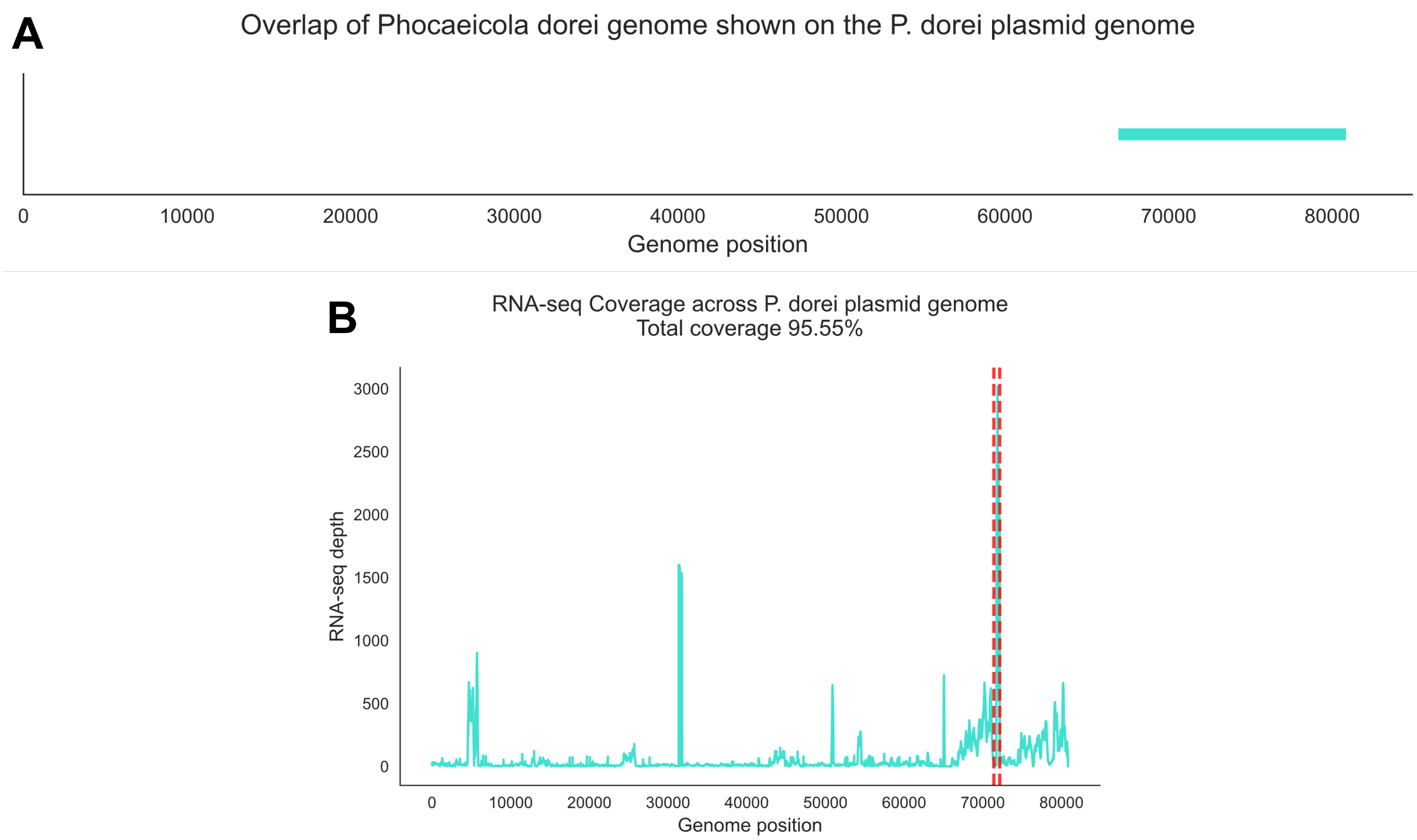

Supplementary Figure 5: Coverage analysis of the plasmid encoding AMP 8200. A) *Phocaeicola dorei* plasmid to genome integration. B) Total coverage of *P. dore*i plasmid genome. The AMP-coding region is highlighted in red.

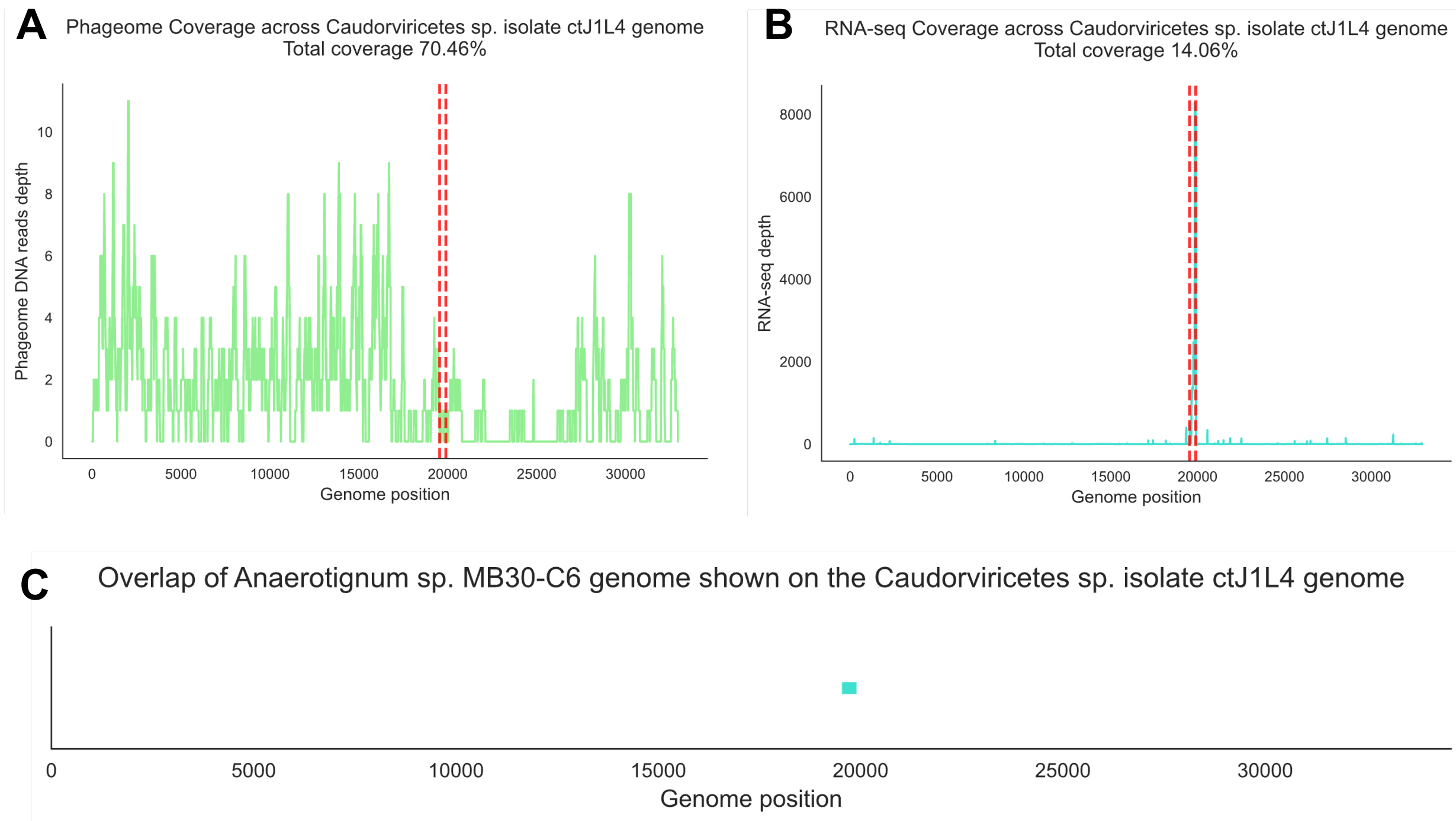

Supplementary Figure 6: Coverage analysis of the phage encoding AMP 3020. A) Phageome read coverage of the Caudoviricetes isolate. B) Metatranscriptome read coverage to the associated Caudoviricetes isolate. The AMP-coding region is highlighted in red. C) Shared region between Anaerotignum sp. and Caudoviricetes isolate.

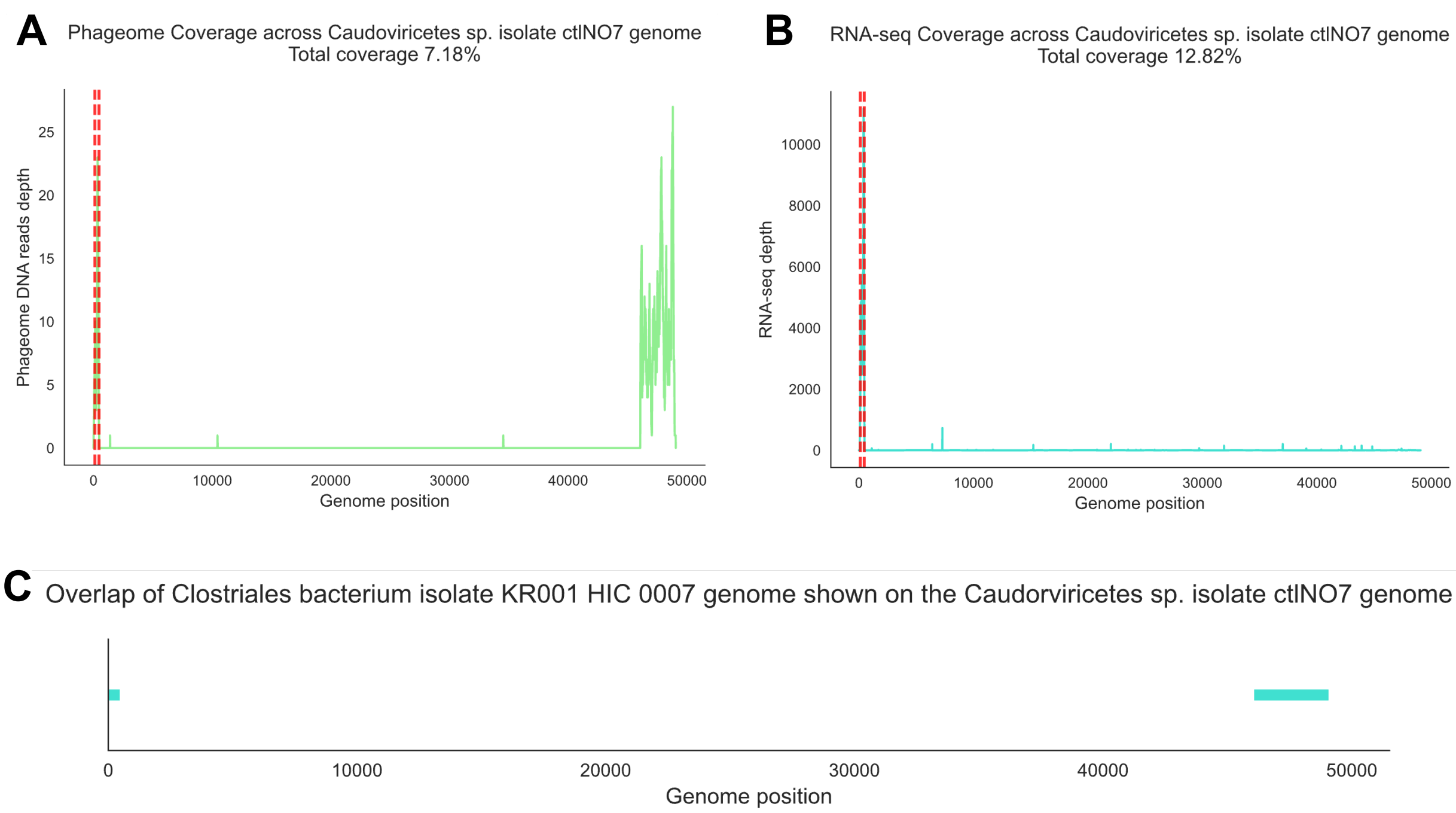

Supplementary Figure 7: Coverage analysis of the phage encoding AMP 8681. A) Phageome read coverage of the Caudoviricetes sp. Isolate ctlNO7. B) Metatranscriptome read coverage to the associated Caudoviricetes sp. Isolate ctlNO7. The AMP-coding region is highlighted in red. C) Shared region between the Clostridiales bacterium and Caudoviricetes sp. Isolate ctlNO7.

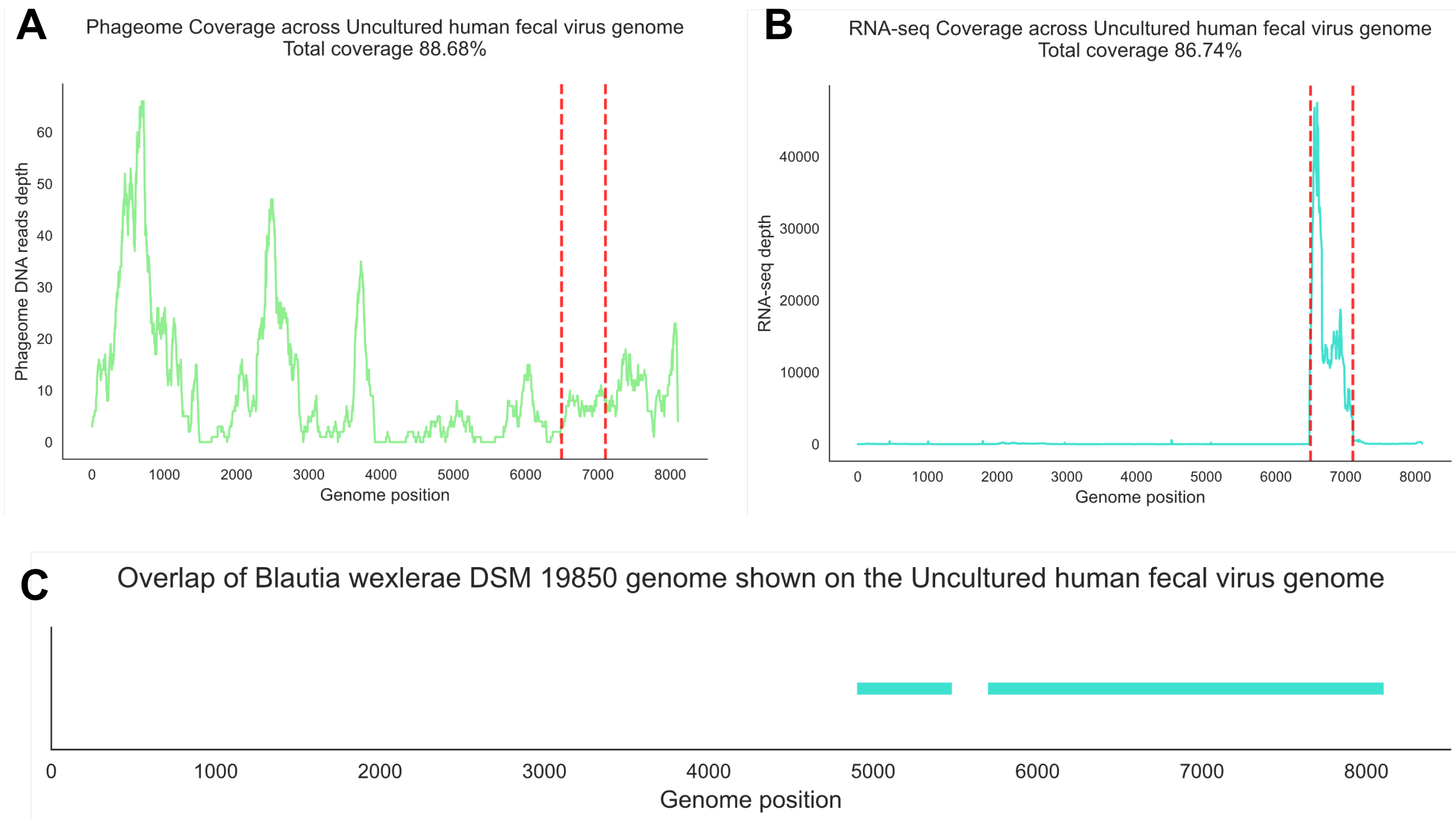

Supplementary Figure 8: Coverage analysis of the phage encoding AMP 5245. A) Phageome read coverage of the Uncultured human fecal virus. B) Metatranscriptome read coverage to the associated the Uncultured human fecal virus. The AMP-coding region is highlighted in red. C) Shared region between *Blautia wexlerae* DSM 19850 and the Uncultured human fecal virus.

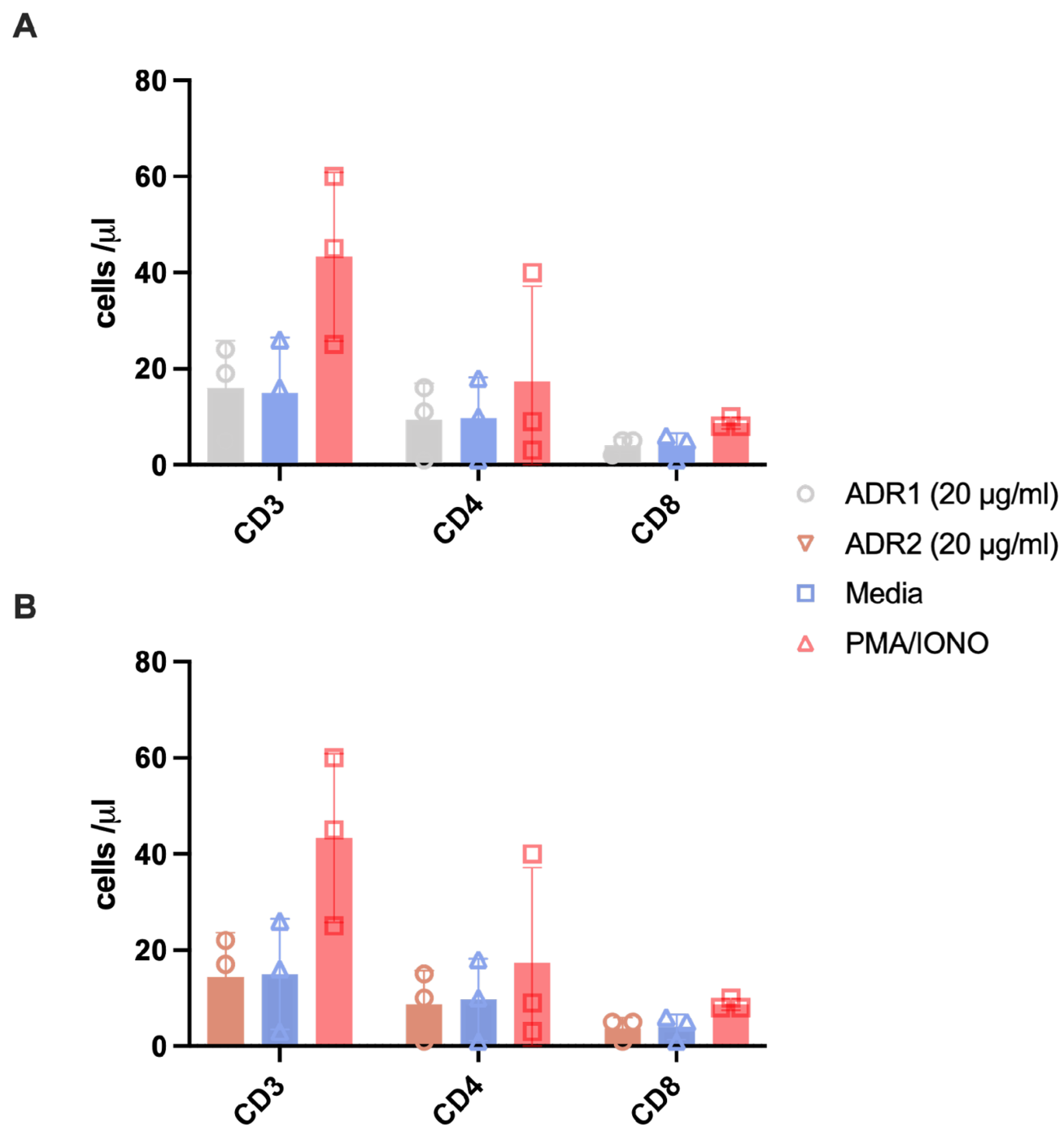

Supplementary Figure 9: Evaluation of the effects of synthetic peptides ADR1 and ADR2 on human T lymphocytes. Absolute number of $CD3^+$, $CD4^+$, and $CD8^+$ T cells following exposure to peptide A) ADR1 and B) ADR2 respectively at (20 µg/mL). No statistically significant differences were observed between peptide-treated groups and the media control.

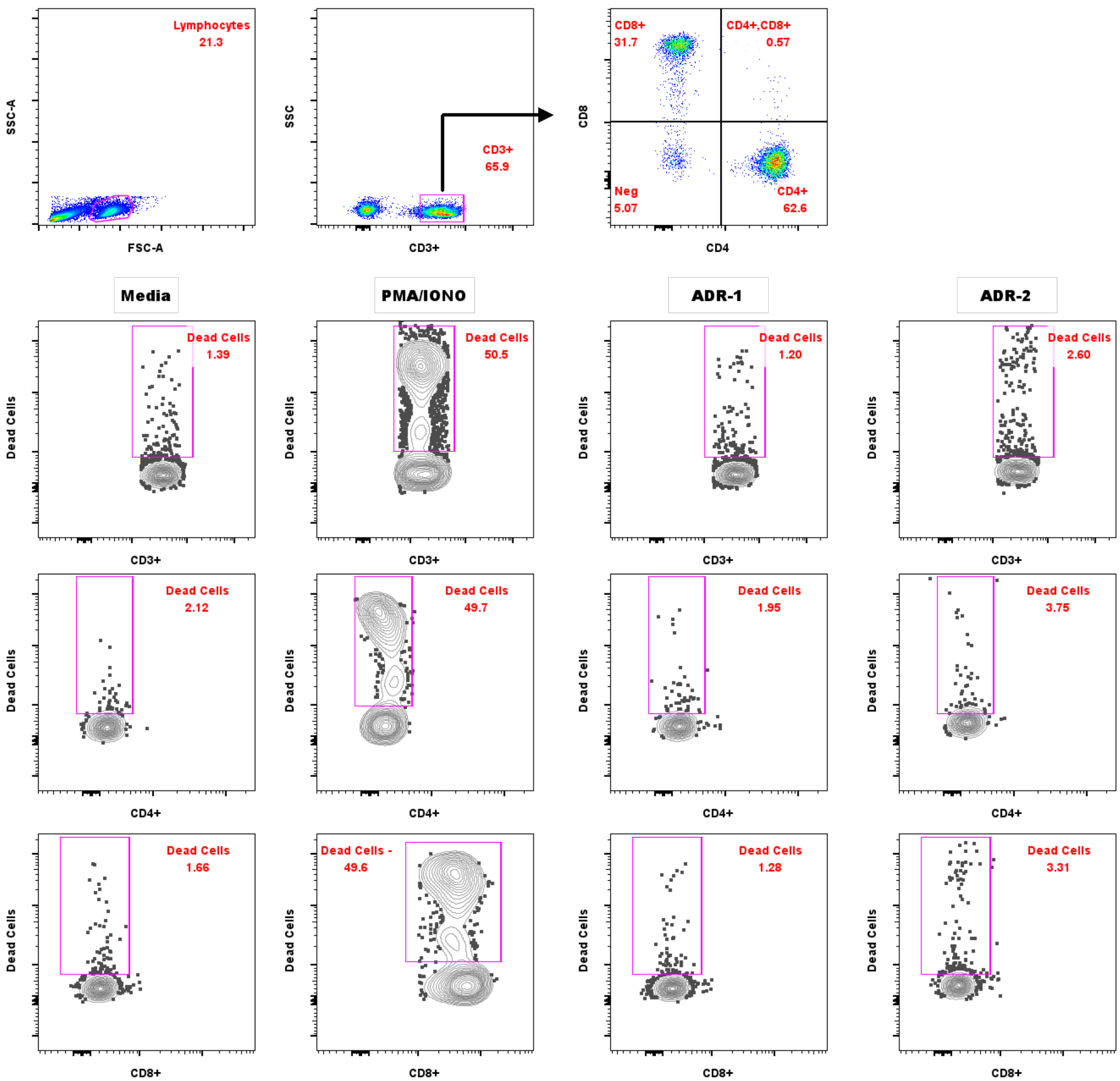

Supplementary Figure 10: Gating strategy for flow cytometric analysis of T lymphocyte viability. Peripheral blood mononuclear cells (PBMCs) were isolated from three healthy donors and stained using the BD TriTest™ reagent to identify CD3⁺ T cells and their CD4⁺ and CD8⁺ subpopulations. Cell viability was assessed using the LIVE/DEAD™ Fixable Near-IR Dead Cell Stain Kit, and absolute cell counts were determined with BD™ Liquid Counting Beads. Representative plots show the sequential gating strategy: initial identification of lymphocytes based on forward and side scatter, followed by gating on CD3⁺ cells, and further discrimination into CD4⁺ and CD8⁺ subsets. The lower panels illustrate the viability of each subset under different experimental conditions: untreated (Media), PMA/IONO stimulation (positive control), and treatment with

synthetic peptides ADR-1 and ADR-2. Flow cytometric acquisition was performed using a BD FACS Canto™ cytometer, and data were analyzed with FlowJo™ software.
